# Re-imagining health professions education in the coronavirus disease 2019 era: Perspectives from South Africa

**DOI:** 10.4102/phcfm.v13i1.2948

**Published:** 2021-08-10

**Authors:** Anna M.S. Schmutz, Louis S. Jenkins, Francois Coetzee, Hofmeyr Conradie, James Irlam, Elizabeth M. Joubert, Dianne Matthews, Susan C. van Schalkwyk

**Affiliations:** 1 Division of Physiotherapy, Faculty of Medicine and Health Sciences, Stellenbosch University, Cape Town, South Africa; 2 Department of Family and Emergency Medicine, Faculty of Medicine and Health Sciences, Stellenbosch University, Cape Town, South Africa; 3 Directorate of Primary Health Care, University of Cape Town, Cape Town, South Africa; 4 Department of Family and Emergency Medicine, Western Cape Department of Health, George Regional Hospital, George, South Africa; 5 Ukwanda Centre for Rural Health, Faculty of Medicine and Health Sciences, Stellenbosch University, Cape Town, South Africa; 6 Division of Human Nutrition, Department of Global Health, Faculty of Medicine and Health Sciences, Stellenbosch University, Cape Town, South Africa; 7 Division of Family Medicine, School of Public Health, Faculty of Health Sciences, University of Cape Town, Cape Town, South Africa; 8 Centre for Health Professions Education, Faculty of Medicine and Health Sciences, Stellenbosch University, Cape Town, South Africa

**Keywords:** COVID-19, health professions education, primary health, responsiveness, remote teaching, clinical training

## Abstract

**Background:**

The coronavirus disease 2019 (COVID-19) pandemic hit South Africa in March 2020, severely disrupting health services and health education. This fundamentally impacted the training of future health professionals and catalysed a significant response from across the health education sector. In 2020, the South African Association of Health Educationalists requested members to submit reflections on different aspects of their COVID-19 related educational responses.

**Responding to the pandemic:**

Seven vignettes focused specifically on clinical training in the context of primary care and family medicine. This short report highlights the key insights that emerged from these vignettes, considering what has been learnt in terms of health professions education and what we need to take forward. These insights include building on what was already in place, the student role, technology in the clinical learning context, taking workshops online, vulnerability and presence and the way going forward.

**Discussion and conclusion:**

The contributions emphasised the value of existing relationships between the health services and training institutions, collaboration and transparent communication between stakeholders when navigating a crisis, responsiveness to the changed platform and dynamic environment and aligning teaching with healthcare needs. It is more important than ever to set explicit goals, have clarity of purpose when designing learning opportunities and to provide support to students. Some of these learning points may be appropriate for similar contexts in Africa. How we inculcate what we have learned into the post-pandemic period will bear testimony to the extent to which this crisis has enabled us to re-imagine health professions education.

## Background

The coronavirus disease 2019 (COVID-19) pandemic hit South Africa (SA) in March 2020 and subsequently more than 1.6 million people contracted the virus.^1^ Lockdown restrictions led to the closing of tertiary educational institutions and forced people to work and learn from home. In health professions education, the disruption fundamentally impacted the training of future health professionals and catalysed a significant response from across the education sector. Those responsible for clinical training had to think creatively and swiftly to navigate the ever-changing situation. Whilst the pandemic continues to wreak havoc, it has created opportunities for reviewing existing practices and approaches to the training of students on clinical platforms. The clinical learning environment, where health professional students work and learn, is foundational to health professions education.^2^ The forced shift to online learning has been extensively documented since the start of the pandemic.^3,4^ Whilst the work that has gone into refurbishing traditional ‘theory’ modules for online engagement cannot be underestimated, sustaining clinical training has provided unique challenges across most health professions.

In July 2020, the SA Association of Health Educationalists (SAAHE) requested members to submit reflections on different aspects of their COVID-19 related educational responses.^5^ Seven of these submissions focused on clinical training in the context of primary health care (PHC) and family medicine (FM). Strategies to strengthen PHC during the global COVID-19 disruption have included service delivery models that promote integrated services, workforce strengthening and use of digital technologies.^6^ In a recent systematic review, Komashie and colleagues point to a diversity of challenges facing healthcare, such as multimorbidity, the complex nature of healthcare delivery and a range of organisational and cultural concerns.^7^ In response, they argue for the adoption of a systems approach to strengthen the quality and delivery of healthcare. Such an approach acknowledges the interconnectedness that exists between all components within the healthcare sector, whilst emphasising the value of adopting adaptive iterative implementation of interventions. Such thinking holds implications for clinical training as well, particularly at a time when the pandemic has exponentially heightened the burden on our healthcare system. We would argue that adopting a systems approach that draws on established relationships with stakeholders, across the health sector, whilst acknowledging cultural and organisational challenges has the potential to effect the adaptive responses that are required at this time.^7^ This short report presents a series of vignettes that describe a range of such adaptive responses; highlighting the key insights that emerged, considering what has been learnt and what needs to be taken forward and reflecting on how, collectively, they represent a systems approach, contributing to strengthening the healthcare in this time of disruption.

## Responding to the pandemic: Seven vignettes

The different contexts, the adaptive iterative implementations during the COVID-19 disruptions and the key insights that were gained are summarised as a series of vignettes in Table 1.

**TABLE 1:** Seven vignettes: Contexts, what was performed and key insights.

No.	Context	What was performed	Key insights
1.	Assessment of undergraduate final year medical students in the longitudinal integrated model (LIM) at Stellenbosch University (SU), Ukwanda Rural Clinical School.^8^ Medical students are placed at district hospitals for clinical training, away from the academic hospital, for the entire final year.	Assessment was adapted from face-to-face case-based assessments to videoconferencing case-based assessments. The students provided a summary of patients whom they managed whilst supervised and this provided a starting point to test their clinical reasoning. Students collaborated in the process of identifying issues with the online assessments and finding solutions.	Hospital and clinic staff acknowledged that whilst having students at their sites added to their responsibilities, the benefits outweigh the challenges.^9^ Assessment training and clear instructions for all examiners was crucial to ensure success in videoconference assessments.^10^ Practical tips include: Have an administrator arrange the assessments and carry out a test run with each of the new examiners to ensure familiarity with the videoconference software. Communicate the time allocation for patient presentation, questioning, consensus scoring and feedback to students and examiners before the assessments.
2.	Human nutrition. A 6-week integrated community-based rotation for final year dietetic students within the Ukwanda framework had to change because students could not be placed at pre-existing sites.	The purpose of the programme, to expose students to practice integrated community nutrition, therapeutic nutrition and food service management, was maintained with a shortened 5 weeks rotation: Three weeks online and two weeks at different locations with a non-governmental organisation (NGO).	Linking with an NGO ensured that the students’ community exposure was retained. Programme coordinators ensured that intended learning outcomes were constructively aligned and retained. Assessment was still guided by the set programme outcomes. Most summative assessments were conducted during the online phase whilst the practical phase consisted of formative assessment opportunities. Students received feedback about their competencies, including consultation, health promotion and education skills from their clinical facilitator, the community and peers. Learning opportunities were adapted for COVID-19 nutrition specific responses, for example, developing, preparing and evaluating a soup recipe at a community-based organisation (CBO), compiling guidelines for emergency food parcels and packing food parcels. Through webinars, students gained exposure to national and international role players from different sectors and disciplines highlighting an integrated approach to health and nutrition.
3.	Postgraduate family medicine training in rural areas such as the Garden Route district in South Africa poses challenges, with registrars spread across large geographic areas. Prior to COVID-19 clinical teaching relied on the local family physician who might not always be available because of clinical responsibilities. This meant that planned teaching did not always occur. Face-to-face interactions within a professional relationship, where communication skills such as active listening, empathy, kindness and respect are inherent, remain an organisational challenge.	Prior to COVID-19 the one electronic tool to support workplace-based training and assessment was the e-portfolio. As COVID-19 has disrupted face-to-face meetings, the e-portfolio, with supervision from a distance, has become an indispensable tool in assessment for learning and assessment of learning. Registrars upload educational activities and supervisors at a distance validate and give feedback. Face-to-face educational meetings and learning conversations now happen in cyberspace. Typical platforms include Microsoft Teams^©^ and Zoom^©^.	The COVID-19 disruption has forced an adaptive iterative implementation of previously less utilised digital technologies, which has now become essential to connect within supervisor–learner relationships over large distances in rural areas.
4.	Since 2016, the University of Cape Town (UCT), Faculty of Health Sciences has offered two elective courses for senior medical students.^11^ The faculty’s COVID-19 response provided the opportunity to offer prescribed electives or ‘selectives’, for academic credit.	With assistance from student societies, students signed up for COVID-19 ‘hotline’ shifts at the provincial Disaster Management call centre, for home-based telephonic case and contact tracing for a hospital-based Health Screening and Testing Centre or for COVID-19 clinical duties. Volunteers were eligible for a UCT community service award, which will show on their academic transcripts. Students made helpful suggestions for orientation, asked for regular evidence updates and further training in handling ‘difficult’ callers, and requested additional COVID-19 selective options in future. Supervisors commended their professionalism, empathy, teamwork and initiative, which included one student having the contact tracing interview guide translated into Afrikaans and isiXhosa.	It was not only the curriculum – the way it was packaged – that had to change. The students themselves took on different roles. The reports and supervisor comments of 10, 5th-year and 16, 6th-year selective students were reviewed. All students reported a deeper understanding about COVID-19 clinical management and the pandemic response, better skills in telephonic interviewing and counselling and excellent teamwork and support from their supervisors. They enjoyed ‘making a difference’, despite long and emotionally draining shifts. They faced the challenges of tracing individuals, of language barriers and of telephonic history-taking and counselling, whilst coping with remote academic learning for the first time. Students described the need to prepare well and to reflect regularly as a coping strategy.
5.	Pre-COVID-19, SU physiotherapy students trained in the traditional rotation-based model, completing short rotations across four core disciplines. The Division of Physiotherapy reframed clinical learning opportunities, student support strategies and assessment. The challenge in adapting clinical learning opportunities was to balance responsiveness to emerging opportunities on the ‘new’ clinical training platform, whilst graduating clinically competent entry-level physiotherapists.	Physiotherapy services were directly affected by the redeployment of the health workforce and suspension of ‘non-essential’ health services and de-escalation of care for patients with chronic conditions. The division adopted ‘the curriculum is the patient that walks through the door’ approach and engaged anew with clinicians, recognising this approach included uncertainty towards available clinical learning afforded to students. The division revisited the core competencies for entry-level physiotherapists and developed a masterplan aligned with the Health Professions Council of SA (HPCSA) recommendations. A clinical referral pathway mobile application, Vula (https://www.vulamobile.com/), to track students’ clinical exposures on the platform and map this in relation to the masterplan was introduced. This process helped lecturers to identify and develop supplementary clinical learning activities to address unattained exposures and engaged students in their own learning. These activities included student-led remote case management discussions and virtual home visits.	The adaptation of the clinical learning model resulted in alternative student support strategies to shift the burden away from clinicians and included remote support from a mentor whose focus was to assist the student in self-directed learning. Site supervisors continued providing bedside supervision whilst academic experts facilitated case-based learning through remote communication. Constructive alignment between learning opportunities and assessment were maintained by introducing case-based management discussions (CBDs) in addition to direct observation clinical evaluations tests.^12^ Case-based management discussions assess the student’s competence to manage care comprehensively through the continuum of multimorbidity and the development of graduate attributes.
6.	The SU network for strengthening rural interprofessional education (SUNSTRIPE) project, one of the partner institutions of African Forum for Research and Education in Health (AFREhealth) and University of California San Francisco (UCSF), embarked on a project called Strengthening Inter Professional Education to Improve human immunodeficiency virus (HIV) Care (STRIPE HIV) across Africa.^13^ The goal of STRIPE HIV is to improve the ability of health professional graduates to deliver high quality, team-based, person-centered care to persons with HIV. A panel of 10 experts, representing faculty members of 9 medical and nursing schools in sub-Saharan Africa (SSA) developed a training package consisting of 17 modules focused on core clinical, public health, interprofessional care and quality improvement (QI) domains related to HIV service delivery. Training was delivered through 2-day interactive workshops emphasising the importance of interprofessional care and a culture of QI.	With the onset of the COVID-19 pandemic a COVID-19 module was developed and presented as an adapted online interprofessional workshop. After trying out different formats and platforms, the online Zoom^©^ platform was used because of its facilities for small group work. More than 600 students and health professionals from various professions have been trained in these 90-min workshops, appreciating the highly interactive engagement with the material and the degree of interprofessionality.	SUNSTRIPE has adapted four of the STRIPE HIV modules to the synchronous online format. Guidelines for facilitating these workshops, including in the Zoom^©^ format, have been developed. Some modules were also adapted to be delivered as asynchronous workshops using Google Classroom providing alternative options for connectivity challenges. Appreciating the complexity of healthcare and organisational challenges during the COVID-19 pandemic, interprofessional education in HIV care across Africa was strengthened through iteratively adapting the project.
7.	The UCT Division of Family Medicine’s undergraduate programme reconsidered the way students learn and are taught.^13^	The undergraduate clinical training programme began with Emergency Remote Teaching and was followed by a blended approach of online learning and experiential clinical service learning. There was pre-engagement with online content, which encouraged students to have enriched clinical exposure whilst gaining valuable experience from the clinical response to a pandemic. Plans include the continuation of the blended learning approach using WhatsApp, Zoom^©^ and Vula^©^ platforms to maximise the time for clinical teaching and to pursue an Integrated Teaching Platform for the rural rotation.	Medical students use an online recorded WhatsApp formative role-play intervention that not only accommodates the needs of the most vulnerable students in poorly resourced, low-tech environments but also seeks to reveal the multiple ways in which students’ tacit knowledge of digital learning is displayed. It is asynchronous and suits both student and teacher in a learning relationship that harnesses self-disclosure and uncertainty yet promotes deeper learning.^11^ This format facilitated authentic, ‘just-in-time’ (need-related) learning experiences and harness elements of vulnerability.^14^

COVID-19, the coronavirus disease 2019.

## Discussion

Clinical training should always be responsive to local healthcare needs to promote learning and complement service delivery.^15^ The restrictions and protocols implemented as a result of COVID-19 were undeniably a catalyst for rethinking the potential use and opportunities afforded through different technological processes and applications. The key health professions education lessons learnt during the COVID-19 pandemic are summarised in Table 2. Although this rethinking came about in pressurised circumstances and often amounted to ‘emergency remote teaching’, we have presented some examples of innovations and lessons learnt that came about during the COVID-19 pandemic, which may provide solutions for others involved in health professions education in the post-COVID era.

**TABLE 2:** Key health professions education lessons learnt during the coronavirus disease 2019 pandemic.

No.	Education lessons learnt during the coronavirus pandemic.
1.	Adapt existing innovations within the clinical training context, such as distributed (remote) platforms for clinical training.
2.	Build on established relationships with stakeholders across the health sector, universities and NGOs.
3.	Retain and constructively align the educational or pedagogical principles that had previously informed practice, including assessment of intended learning outcomes.
4.	Adapt learning opportunities for COVID-19 specific responses, for example, nutrition needs of communities.
5.	Webinars allow students to gain exposure to national and international role players from different sectors and disciplines.
6.	E-portfolios overcome logistical challenges by allowing registrars to upload educational activities for supervisors at a distance to validate and give feedback, supplemented with online educational meetings and learning conversations.
7.	The roles of students changed, where the teacher–student relationship is seen as a partnership of co-learning and risk taking, allowing opportunities for development of professionalism, empathy, teamwork and initiative, welcoming self-disclosure, uncertainty and failure to deep learning.
8.	Rethink the potential use and opportunities afforded through different technological processes and applications, such as the clinical referral pathway mobile application, Vula^©^, to track student exposures on the platform and using videoconferencing in student assessments.
9.	Online platforms such as Zoom^©^ allow small group modular work to be conducted in workshops through highly interactive engagement with material and interprofessionality on a global level.
10.	Applications such as WhatsApp allow exercises in which students use an online recorded WhatsApp formative role-play intervention that accommodates the needs of vulnerable students in poorly resourced, low-tech environments and also seeks to reveal the ways in which students’ tacit knowledge of digital learning is displayed. More individual contact (via WhatsApp) between students and trainers represent learning around patient encounters in the daily clinical workspace.

NGOs, non-governmental organisations; COVID-19, the coronavirus disease 2019; No., number.

Existing relationships between health institutions and the clinical service platform that have developed over many years have been invaluable in rapidly adapting the clinical environment to the global pandemic.^16^ This emphasises the need for health professions education to continue training in practical settings at grassroots level, close to people and communities. These new circumstances have highlighted the value of providing opportunities for students to be exposed to practical integrated healthcare and to engage with a much broader scope of platforms offered by, for example, non-governmental organisations (NGOs) and community-based organisations (CBOs). It can be argued that the COVID-19 disruption has enabled adaptive iterative implementation of previously less-utilised digital technologies, such as electronic learning portfolios (e-portfolios), Zoom^©^ and Vula^©^, which have now become essential to connect within supervisor–learner relationships over large distances in rural areas.^17^ In addition, the pandemic has provided an opportunity for a more student-centred approach that sees the teacher–student relationship as a partnership of co-learning and risk taking – one that welcomes self-disclosure, and acknowledging uncertainty and failure as pathways to enrich learning.^14^ The pandemic is ‘an opportunity to not only rethink online digital pedagogies but also to reimagine education …’ where creating new, intersecting relationships, new forms of learning and a new respect for different modes of knowledge is valued to create more equitable, humane and just societies.^18,19^

## Conclusion

Over the past year, clinical educators across SA have collectively demonstrated their ability to adapt using innovative approaches whilst drawing on established, pre-existing relationships and whilst navigating the complex healthcare system. Much of what has happened has been influenced by technology. Online meetings have allowed participants (students, educators and clinicians) from far and wide to engage, without barriers of cost, travel or losing travel time. This has become a new way of collaborating, enabling participation across geographical borders, exposure to leaders in the field and disciplinary experts engaging one another. Whilst there previously might have been a reluctance by many clinical educators to move teaching and learning activities into the online learning environment, the combination of existing online activities and the pressures to increase their use that came with the COVID-19 pandemic allowed for a rapid adoption of these new modes of teaching. The COVID-19 crisis has emphasised the value of collaboration and communication between stakeholders in adapting to a changed clinical and learning platform and aligning teaching with community healthcare needs.
